# Rheumatic Fever and Its Treatment

**Published:** 1909-01-02

**Authors:** William Murrell

**Affiliations:** Physician to the Westminster Hospital, and Joint Lecturer on Medicine and Clinical Medicine


### Hospital Clinics.

### RHEUMATIC FEVER AND ITS TREATMENT.

By WILLIAM MURRELL, M.D., F.R.C.P., Physician to the Westminster Hospital, and Joint Lecturer on Medicine and Clinical Medicine.

(A Clinical Lecture delivered at the Westminster Hospital.)

Acute rheumatism or rheumatic fever is a common disease both in children and adults. It is an acute specific fever of long duration (when untreated), with a special proclivity for the joints and the structures of the heart. It is a disease of all temperate climates, but is especially prevalent in Great Britain. It sometimes assumes an epidemic form, and one attack predisposes to others. In London the majority of cases are seen in the autumn and winter months, October as a rule marking the opening of the rheumatic as of the enteric season. In adults most cases occur in young men, probably because they are more liable than women to exposure to cold and wet. Below the age of ten it is equally common in girls and boys, but from ten to fifteen there are more cases in girls, possibly because of their greater liability to attacks of chorea. There is a belief that it is hereditary, but there is little evidence to support this view; at all events, heredity is not an important ætiological factor. It is an occupation disease in the sense that certain trades and callings involve exceptional liability to exposure. It is not contagious and it is not infectious, although the recurrence from time to time in quick succession of a number of cases in the same house would seem almost to throw doubt on this assertion. It affords no protection or immunity; on the contrary one attack predisposes to others, and it is not uncommon to meet with patients who have suffered from half a dozen or more attacks, some severe and others comparatively trivial.

There is little doubt that rheumatic fever is microbic in origin, its especial proclivity for children, its general course, and its analogy to the acute specific diseases favouring this view. What may be the nature of this organism is open to doubt. The general consensus of opinion was until recently in favour of the diplococcus of Paine and Poynton—the diplococcus rheumaticus—which is apparently identical with the diplococcus discovered by Tribolet in 1897 and described by Wassermann in 1899. This micro-organism is very minute and is by no means easy to demonstrate. "It is a micrococcus and yet a streptococcus, because it may grow in chains; a diplococcus because its elements are usually coupled; and a staphylococcus because in solid media it may grow in bunches." Pure cultures have been obtained from the vegetations on the mitral valve and from the spleen and kidneys. The intravenous injection of the diplococcus usually, although not invariably, produces in rabbits and monkeys arthritis, valvulitis, and pericarditis, but it is by no means clear that this condition is identical with the rheumatic fever of the human subject. Not being a bacteriologist I ought perhaps to refrain from offering an opinion, but it seems to me that this particular organism has been losing favour of late; at all events both in Paris and in Glasgow, where extensive investigations have been made with it, it has lost ground.

Meyer describes a strepto-diplococcus which injected into rabbits produces arthritis with effusion followed by endocarditis and pericarditis, and there is also a bacillus discovered by Achaline in 1891 similar in appearance to that of anthrax. It is found not only in the blood but in the cerebro-spinal fluid. It can be cultivated without difficulty, and when grown in sterilised urine throws down a copious precipitate, said to be more marked in the urine of arthritic than in that of healthy people. Sodium salicylate even in dilute solutions arrests its growth. In experimental inoculations it was found associated with streptococci in the serous fluids, an indication that the microbe opens the door to the microbes of secondary infections. Staphylococci and streptococci have been found in the synovial fluid of the joints, in the pericardial fluid, and in connection with the valves, but these may have been cases of ulcerative endocarditis.

The present position of affairs seems to be this, that one or more organisms having distinctive characters have been isolated from typical cases of rheumatic fever, cultivations of which, when injected into certain animals, have produced symptoms like those of rheumatism, and have then been recovered from the infected animals. Streptococci and staphylococci will not produce these effects even when their virulence has been intensified. It is claimed that the micrococcus rheumaticus bears the same relationship to acute rheumatism that the pneumococcus does to pneumonia; that it is one of the causal agents in the production of rheumatism although not the only one.

The old nervous and metabolic or chemical theories of acute rheumatism are exploded, and are not worth discussing, whilst Richardson's lactic-acid theory has long been discarded.

The onset of an attack of acute rheumatism is usually moderately abrupt, following some definite exposure to cold and wet. In one case within my recollection it occurred from sleeping in an attic, the bed being placed close to a large cistern of cold water; and in another it resulted from a young man, whilst in a state of intoxication, going to bed with a wet umbrella. Sore-throat is often the first symptom, and the association of tonsillitis with rheumatic fever is well recognised. From throats of this character a diplococcus has been isolated, which on intravenous inoculation into rabbits produces endocarditis and arthritis, a diplococcus identical with that previously isolated from and demonstrated in rheumatic lesions. Many attacks of tonsillitis are not followed by rheumatic fever, whilst a particular attack in the same individual may have this sequence. In a large proportion of cases the site of entrance of the specific organism of acute rheumatism is the tonsil. The temperature as a rule is not high, ranging usually from 101° to 104°, except in those somewhat infrequent cases in which there is hyperpyrexia. The routine use of cold or tepid sponging when the temperature reaches 105° probably accounts for the comparative rarity with which high temperatures are seen. It is said that the severity of the fever depends on the number of joints simultaneously involved, but I cannot confirm this statement. The middle-sized joints, such as the knee, wrist, and ankles, are those most commonly involved. The joint affected is hot, red, swollen, and tender, and there may be some effusion, although it is very rarely much. Metastasis is often seen, the inflammation disappearing in one joint as the corresponding joint on the opposite side becomes involved. There is, of course, no actual transference of inflammation, and metastasis simply means that corresponding joints are affected in succession. The same joint is rarely inflamed for more than four days. The affection is nearly always polyarticular, and when only one joint is involved some other cause than rheumatic fever should be suspected. The joints of the lower extremities are the most frequently attacked, but when the patient's occupation involves hard manual labour the wrist, elbow, or shoulder may be the first to suffer. A good example of this is seen in electric-tram drivers, who are well covered with regard to the lower extremities and use their arms almost exclusively for their work. It is probable that the pain experienced is not so much in the joints themselves as in the adjacent muscles and tendons.

Sweating is another fairly constant symptom, although the sweat is not of necessity acid in reaction, and the peculiar acrid odour often described is not commonly observed. When sweating is profuse sudamina and miliaria are generally to be seen on the chest, especially when the skin is looked at sideways. Of other rashes met with in rheumatic fever a specific erythema papular in form is described, but it is certainly not common; and it will be remembered the salicylates produce skin eruptions, usually either an urticaria or an erythema. In connection with rheumatic fever, erythema nodosum, and peliosis rheumatica are often seen. There are, in addition to the specific symptoms, the usual symptoms common to all febrile affections, fever, frequency of pulse, headache, muscular pains, thirst, furred tongue, loss of appetite, constipation, and urine of high specific gravity loaded with urates. Anæmia is marked, the number of red cells being considerably diminished, in cases of short duration to 4,000,000 per c.mm., and in prolonged cases by another million. In some cases it may be as low as 1,250,000. The leucocytes are usually about twice the normal, but may rise to 30,000 per c.mm. in severe attacks. The micro-organisms gain access to the heart from the tonsils by the blood-current, multiplying on tissues favourable to their reception and growth. The valves most frequently affected are the mitral and the aortic; the tricuspid is sometimes damaged, the pulmonary rarely. Dilatation of the heart is common, and the first clinical evidence of heart damage is often myocardial rather than endocardial. Dilatation may occur independently of endocarditis and pericarditis. Delirium and coma are met with in a comparatively small percentage of the cases. Myocarditis is responsible for the cases of sudden death met with in this affection.

The connection existing between scarlet fever, tonsillitis, acute rheumatism, chorea, pericarditis, myocarditis, endocarditis, and certain other diseases has long been known. A common sequence is tonsillitis, rheumatic fever, chorea, pericarditis, and endocarditis. Sometimes chorea comes first, and scarlet fever may take the place of rheumatic fever, or may be followed by it. Whether chorea is microbic in origin is still undetermined, but in some cases it has been attributed to staphylococcus aureus, and it is quite possible that the aureus infection may be one of the factors in the production of the acute form of the disease.

Stockman recognises two forms, or perhaps varieties of acute rheumatism; one corresponding to that just described which is amenable to the salicylates, and another but little influenced by their administration, which attacks chiefly the fibrous tissues and runs a much more chronic course.

The history of the treatment of rheumatic fever presents some points of interest. In the eighteenth century the remedies commonly employed were venesection—often to 70 ounces frequently repeated—leeching, tartarised antimony, antimonial powder, the compound powder of ipecacuanha, and cicuta, the last being the old Roman name for hemlock or conium. In 1805 John Haygarth published a number of cases treated with cinchona or Peruvian bark at the suggestion of Dr. John Fothergill and Sir Edward Hulse, who is described as the then most eminent physician in London. The bark was given in various forms; as a powder in doses of from five grains to a drachm, as the decoction in doses of one to two ounces, and also as the tincture. It appears that out of 170 cases 11 died, and that out of 25 cases 22 were "restored to health" in 30 days, which hardly compares favourably with modern practice, especially as one case lasted from 30 to 40 days, another from 40 to 50, and a third from 70 to 80. From a careful examination of his paper I am inclined to think that the author was not always accurate in his diagnosis and that in some instances he confounded malarial infection with rheumatic fever; at all events the following quotation is suggestive: "From so many circumstances of similarity between the ague and the rheumatick fevers both as to their symptoms and their remedies some have supposed them to be the same disease." For how long this cinchona treatment held sway I do not know, but it seems after a time to have been superseded by calomel and opium. The opium may have been of some use in allaying the joint pain and promoting sleep, and the calomel would neutralise its constipating effects, but the combination could have exerted no real action in arresting or shortening the duration of the disease. This was ultimately supplanted by the alkaline treatment advocated by Fuller and others, and for many years was adopted by the great majority of medical men in this country, although it found but little favour on the continent. The drug most commonly given was the bicarbonate of potassium and it is stated that the duration of the disease under full alkaline treatment was 35 days. Here, however, we are met with a little difficulty, for in those days no temperatures were taken, and physicians were by no means in accord as to what should be regarded as the terminating point of the disease—some being satisfied to regard the patient as well as soon as the tongue was clean and the pains in the joints had subsided. No value can be ascribed to the treatment of Owen Rees, which consisted in the daily administration of from three to eight ounces of lemon juice on the mistaken theory that it is a vascular stimulant; nor did the substitution of lime juice give better results. It was of no more value than the mint-water treatment.

My first personal experience of the systematic treatment of acute rheumatism was in the early seventies at University College Hospital under Russell Reynolds, who always gave tincture of perchloride of iron in drachm doses every four hours. Whether he believed in this form of treatment I do not know, for he was a reticent man who rarely communicated his views to his subordinates, and I am not aware that he published his statistics. As far as my memory serves me the cases ran their ordinary course.

My next experience was at the Westminster Hospital where Basham—whose name is chiefly remembered in connection with Basham's mixture—treated his cases with nitrate of potassium, a drug introduced in this connection by Brocklesby nearly 100 years previously. Basham published a paper on the subject in the "Transactions of the Medico-Chirurgical Society," from which it appears that the average duration of the illness in 56 males was 31 days, and in 20 females 34 days.

I remember perfectly well when the salicylic-acid treatment was introduced about 1876, and the dominant impression on my mind is that in consequence of the impurity of the drug most of the patients were wildly delirious and passed black urine. Salicylic acid had been known to chemists for nearly 50 years when, in 1874, Kolbe succeeded in making it from carbolic acid. He showed that it is a powerful anti-putrefactive and anti-fermentative, and that it has the power even in very small quantities of inhibiting the action of yeast, pepsin, and other ferments. Buss, when investigating its action in various febrile conditions, noted that it is especially useful in acute rheumatism, a conclusion confirmed by Stricker and others. In 1876 Maclagan, then of Dundee, pointed out the wonderful efficacy of salicin, a drug used by him since 1874 in the treatment of the disease. This was the starting-point of the modern treatment of rheumatic fever. The salicyl group is a large one, numbering some forty or more compounds, of which salicin and salicylic acid are amongst the best known. Salicinum, or salicin, is not an alkaloid, but a glucoside obtained from various species of willow. In the body it undergoes decomposition into glucose and salicylic acid, so that its action is that of the salicylates. Its conversion is slow, and it is generally regarded as being inferior in activity to salicylic acid. It is excreted by the urine as salicin, salicylic acid, and salicyluric acid. There are two forms of salicylic acid, the "natural" and the "artificial," the former prepared from salicin or from the essential oils of winter green and sweet birch, and the latter by the interaction of sodium carbolate and carbon dioxide. The artificial acid at first supplied was very impure, containing a considerable proportion of the cresols. Ultimately these impurities were eliminated, and a "Salicylic Acid, Physiologically Pure" was obtained said to be identical with the natural product. This pure acid, being much cheaper, has practically superseded the variety prepared from oil of wintergreen. There are, however, many good clinical observers who maintain that their therapeutical actions are not identical. At all events, in obstinate cases of acute rheumatism, and they are not many, which are "rebellious" to the pure artificial acid it is not a bad plan to try the natural product. Salicylic acid is eliminated rapidly, so that even after large doses it in a few hours cannot be detected in the urine. Salicylate of sodium is the popular member of the group, but is somewhat slower in its action than salicylic acid. Salol or phenyl salicylate is nearly insoluble in water, and its action is due to the fact that it is decomposed in the duodenum into phenol and salicylate of sodium. Aspirin, a fancy name for acetyl-salicylic acid, is extensively used, and is likewise decomposed in the duodenum with the liberation of salicylic acid. There are dozens of other members of this group all more or less efficacious, and it is probable that the majority of medical men, without investigating the subject, prescribe that particular form which is most persistently brought under their notice. It may be of interest to those practising in remote country districts or in the Colonies, far removed from access to drugs, to note that the common meadow-sweet and other species of spiræa, which grow everywhere, contain salicylous acid, another member of this group, and that a decoction or an infusion of the flowers is an excellent remedy for acute rheumatism. Respecting the mode of action of this particular group of remedies we have no real knowledge. It does not advance matters very much by saying that they exert a "specific" action, in the absence of any certain knowledge respecting the specific microbe of rheumat[illegible]c fever. We do not know whether [illegible] amelioratio[illegible] of the symptoms is due to an antitox[illegible] or to anti-[illegible]acterial substance generated in the body. We have found the remedy for the disease without being very positive as to its causation.

I remember the time when the wards were always full of rheumatic fever cases, when the patients groaned or cried out in their agony, and when they displayed the greatest anxiety to prevent the students coming near to or shaking their beds. Now I practically never see an acute case. The patient is admitted perhaps on Saturday night, is put to bed and given 20 grains of salicylate of sodium every four hours. By Monday afternoon the temperature has fallen to normal, the pain in the joints has subsided and the patient is convalescent. The temperature chart is not unlike that of an acute pneumonia, even to the extent that under the influence of the drug the termination may be by crisis or by lysis. Rheumatic fever is especially the domain of the therapeutist, or, as some would say, of the empiricist, and the services of the bacteriologist are not called into requisition. There is not even an agglutination test such as we have in enteric fever. The salicylates are perfectly satisfactory in these acute cases, and it is to be regretted that we have no equally efficacious remedies for the other acute specific fevers. Their sphere of action is confined to acute rheumatism, and to such an extent is this the case that many people regard its prompt action as a test of correctness of diagnosis. It is useless in pyæmia and other conditions which might at a cursory glance be mistaken for rheumatic fever. Whether in course of time the salicylate may be superseded by some other remedy more suitable for the less typical and more chronic forms of the disease remains to be seen, but for the acute cases nothing is likely to supplant it.

It is noteworthy that we have now no positive information respecting the natural course of the disease, for every case is actively treated. Is it possible that in course of time it has undergone some modification? When there was no known remedy Gull and Sutton's observations with mint-water were justifiable, but in the present state of our knowledge we should incur a serious responsibility in withholding from the patient a drug of such recognised value as the salicylate simply with the view of adding to our stock of information.

When salicylates are given in sufficient dose in an uncomplicated case of rheumatic fever, the pain in the joints and the temperature begin to be favourably affected at once, the former subsiding in twelve to twenty-four hours and the latter within forty-eight hours. The joint swelling disappears in two to four days, and the frequency of pulse and respiration diminishes with the fall of temperature. The course of events closely resemble a crisis in fever.

The salicylate of sodium is the salt most commonly employed, and although there is much talk about its depressing action on the heart no practical inconvenience results from its administration. I see no advantage in combining the salicylate with alkalies. Holding this view, I think that it is a decided advantage that aspirin cannot be administered in combination with bicarbonate of sodium, for it prevents prescribers from falling into vicious habits of poly-pharmacy.

It must be admitted that in a certain proportion of cases the salicylates fail to act satisfactorily, and that there may be a relapse or even an exacerbation of the symptoms. Some of these failures may be due to faulty or imperfect diagnosis. But excluding these, some cases run a subacute course and fail to respond readily to treatment. The explanation is probably to be found in the fact that the drug is administered in insufficient doses and that the physician is hampered by his knowledge of the pharmacopœia. It should be remembered that the doses given in the British Pharmacopœia are meant for general guidance but are not "authoritatively enjoined." The pharmacopœial dose of salicylic acid is from 5 to 20 grains and of salicylate of sodium from 10 to 30 grains. When 30 grains of salicylate of sodium every four hours fail to afford relief 40 grains should be given, and repeated if necessary until the temperature and symptoms subside.

In cases of rheumatic hyperpyrexia and in ulcerative endocarditis the salicylates are practically useless, but these complications are treated by other means; by cold sponging in hyperpyrexia and the use of a serum in ulcerative endocarditis.

A special feature of the acute rheumatism of children is the occurrence of subcutaneous nodes or nodules. It is said that they were originally described by Thomas Hillier, of University College Hospital. He died in 1868, and neither in his "Diseases of Children" (1868) nor in his "Handbook of Skin Diseases" (1865) have I been able to discover any reference to the subject. The question of subcutaneous nodules connected with the fibrous structures occurring in children the subjects of rheumatism and chorea was first brought prominently forward in a paper by Thomas Barlow and Francis Warner in 1881, although there had been previous communications by Meynet, Rehn, Hirschprung, of Copenhagen, and by Trosier and Brocq. These nodules vary in size from a pin's head to that of an almond, and they are always subcutaneous. As a rule, they give rise to no pain but sometimes tenderness may be elicited on pressure. The back of the elbow, the malleoli and the margins of the patella are the favourite sites, but they are also found close to the vertebral spines, the spine of the scapula, the crista ilii, the extensor tendons of the foot and hand, the temporal ridge, the forehead and the superior curved line of the occiput. They appear in crops and are usually ephemeral, each crop, as a rule, persisting only a few days. Microscopically, they consist of wavy strands of fibrous tissue with caudate, spindle-shaped nucleated cells and abundant vessels. There is no doubt that they are the result of rheumatic inflammation, and their clinical significance is derived from the indication they afford of similar changes taking place in the endocardium and pericardium so that their occurrence may be regarded as a bad omen.

They are similar to but not identical with the indurations or thickenings which Stockman describes as occurring in the fibrous tissues in the chronic rheumatism of adults. The latter are well defined and circumscribed, varying in size from a small shot or split pea to an almond or even half a walnut. Sometimes they are deeply placed, and at others quite superficially in the subcutaneous tissues. When pressed upon they give rise to pain, especially when the branch of a nerve is involved. They consist of fibrous tissue and an amorphous sero-fibrinous matrix. Fibro-blasts are not numerous, and there is no congregation of leucocytes in the neighbourhood of the inflamed area. Dr. Stockman tells me that he does not think that they are identical with the form met with in children, seeing that they are more or less permanent and not ephemeral or evanescent as are those described by Barlow and his colleague. There are many other different kinds of nodes and nodosities, but I do not think that either of these forms could be confounded with Heberden's nodes or with Haygarth's nodosities. Moreover, they are quite distinct from gouty tophi; and from Parrot's nodes, which are due to periostitis probably syphilitic in origin.

